# Cause for concern in the use of non-steroidal anti-inflammatory medications in the community -a population-based study

**DOI:** 10.1186/1471-2296-12-70

**Published:** 2011-07-07

**Authors:** Robert J Adams, Sarah L Appleton, Tiffany K Gill, Anne W Taylor, David H Wilson, Catherine L Hill

**Affiliations:** 1 The Health Observatory, University of Adelaide, The Queen Elizabeth Hospital Campus, Woodville Road, Woodville, SA, Australia; 2 Population Research & Outcomes Studies Unit, The University of Adelaide, Adelaide, SA, Australia; 3 Rheumatology Unit, The Queen Elizabeth Hospital, Woodville Road, Woodville, SA, Australia

**Keywords:** COX-2 inhibitors, Non-steroidal anti-inflammatory, chronic disease, cardiovascular risk

## Abstract

**Background:**

Non-steroidal anti-inflammatory (NSAID) medications are a common cause of reported adverse drug side-effects. This study describes the prevalence of non-steroidal anti-inflammatory (NSAID) use (other than low-dose aspirin) and the presence of co-existing relative contraindications to NSAID use and chronic conditions in a representative population sample.

**Methods:**

Data were analysed from 3,206 adults attending first follow-up of the North West Adelaide Health Study (NWAHS) in 2004 - 2006, a longitudinal representative population study. Medications were brought into study clinic visits by participants. Clinical assessment included measured blood pressure, kidney function, serum cholesterol, blood glucose. Questionnaires assessed demographics, lifestyle risk factors, physician-diagnosed chronic conditions. Data were weighted to census measures by region, age group, gender, and probability of selection in the household, to provide population representative estimates. Pearson's Chi-square tests determined significant differences in proportions. Multiple logistic regression was used to examine associations of socio-demographic characteristics with use of NSAIDs.

**Results:**

Of 3,175 participants, 357 (11.2%), and 16% of those aged > 55 years, reported using either non-specific NSAIDs or COX-2 inhibitors, other than low-dose aspirin. Among people using NSAIDs, 60.8% had hypertension, 30.8% had Stage 3 or higher chronic kidney disease, 17.2% had a history of cardiovascular disease (CVD) and 20.7% had a > 15% 10-year CVD risk. The prevalence of NSAID use among people with hypertension was 16%, with kidney disease 15.9%, and a history of CVD 20.0%. Among people taking diuretics, 24.1% were also taking NSAIDs, and of those taking medications for gastro-esophageal reflux, 24.7% were on NSAIDs. Prescription-only COX-2 inhibitors, but not other NSAIDs, were used more by people > 75 years than by 35-54 year olds (OR 3.7, 95% CI 2.0, 6.7), and also were more commonly used by people with hypertension, cardiac and kidney disease.

**Conclusions:**

There is a high prevalence of current NSAID use among groups at-risk for significant drug-related adverse events or who have major chronic conditions that are relative contraindications to NSAID use. Assessment of absolute risks regarding cardiovascular and kidney disease need to take into account use of medications such as NSAIDs. The potential to make a substantial impact on chronic disease burden via improved use of NSAIDs is considerable.

## Background

Non-steroidal anti-inflammation drugs (NSAIDs) are one of the most common causes of reported serious adverse reactions to drugs, with those involving the upper gastrointestinal tract (GIT) [1], the cardiovascular system [2,3] and the kidneys being the most common [2,3]. Much of the focus on NSAID adverse effects has been on GIT consequences, with good reason. A US study found the rate of deaths from NSAID-related GIT adverse effects is higher than that found from cervical cancer, asthma or malignant melanoma [4]. Significant deterioration in blood pressure [5], development of chronic heart failure (CHF) [6] and serious cardiovascular events can also occur with a number of NSAIDs [7]. Risk is increased among the elderly and those with co-morbidities [2,3]. It has been suggested that the "burden of illness resulting from NSAID-related CHF may exceed that resulting from GIT damage" [6]. Recent evidence from a Danish population study suggests that cardiovascular mortality is increased among people without a prior history of cardiac disease by NSAIDs, particularly diclofenac and ibuprofen [8]. However, the baseline cardiovascular risk of people in this study was not reported.

NSAIDs are implicated in rapid deterioration of renal function [9,10], so national guidelines recommend the avoidance of nephrotoxic drugs (including NSAIDs) in people with chronic renal impairment [11].

Few studies in Australia have examined the frequency of NSAID use among population groups at-risk for adverse events or significant drug interactions. An audit of rural general practices in Queensland found risk factors for NSAID-related adverse events (mostly older age, hypertension and previous peptic ulcer disease) in 65.1% of patients prescribed COX-2 inhibitors [12]. A claims database analysis found use of NSAIDs in around 15% of those taking diabetes medications [13]. These studies have been unable to assess the effect of NSAIDs on measures such as blood pressure or cardiac events in these at-risk groups, particularly post-October 2004 when rofecoxib was withdrawn from the market. The aim of this paper was to describe in a representative population sample the prevalence of NSAID use (other than low-dose aspirin), including differential use of COX-2 inhibitors and non-specific NSAIDs (ns-NSAID) and the presence of co-existing contraindications to NSAID use and chronic conditions (including cardiac/cerebrovascular disease, hypertension, chronic kidney disease, diabetes, and high 10 year-risk for cardiovascular disease).

## Methods

The NWAHS is a representative biomedical population study of people aged ≥ 18 years randomly selected from the electronic white pages telephone directory and living in the north western suburbs of Adelaide, South Australia. The distribution of social indicators is representative of the community profile of Adelaide [14]. The methods for NWAHS [14] and the ability of these methods to achieve an unbiased population-based sample have been described previously [15]. In 2000 - 02, 4,060 people attended the clinic for biomedical examination, including surveys and clinical measurements of blood pressure, height, weight, waist and hip circumference, fasting glucose and lipid levels [14]. In 2004-2006 all participants were recontacted, with 3,522 completing interviews (86.7%), of which 3,206 (79.0%) attended for clinic assessment, 120 (3.0%) died or were too ill to attend, 92 (2.3%) were unable to be contacted, and 326 (8.0%) declined to participate. Compared with those who did not complete reassessment in the clinic, persons who remained in the study were not significantly different across the study variables in terms of demographic characteristics or disease distribution. This analysis was conducted in 3175 subjects who provided medication data.

Information was collected on health behaviours (smoking, alcohol use), doctor diagnosed health conditions, symptoms of joint pain (in at least one of the following sites - foot, knee, hip, hand, shoulder), health service utilization and demographics. All current medication use was recorded as dose, brand, type and usage pattern of all medications including prescription, over-the-counter and complementary medications that participants were currently using and were presented at the clinic visit. The study was approved by the North West Adelaide Health Service institutional ethics committee, and all subjects gave written informed consent.

### Risk factor and Chronic Disease definitions

*Hypertension*: either systolic ≥ 140 mm Hg and or diastolic ≥ 90 mm Hg, self-reported physician diagnosis or on medication. *High cholesterol*: fasting total cholesterol ≥ 5.5 mol/l. Coronary Heart Disease (*CHD*): self-reported physician diagnosed myocardial infarction or angina. *Stroke*: self-reported physician-diagnosed stroke or cerebrovascular event. *Diabetes*: fasting blood glucose ≥ 7.0 mmol/l, self-reported physician diagnosis of diabetes, or treatment for diabetes. *Chronic kidney disease*: Glomerular filtration rate (GFR) was calculated using the Cockcroft-Gault equation and kidney disease was defined as: GFR < 60 ml/min/1.73 m^2^, or GFR > 60 and proteinuria and was defined as Stage 3 or higher (GFR < 60), according to national guidelines [11]. *Joint pain: *doctor diagnosed conditions (including arthritis [osteoarthritis, rheumatoid arthritis or other arthritis]) and undiagnosed joint pain was defined as pain in at least one site that had not been previously classified by a doctor. *High risk for Cardio-vascular disease (CVD)*: 10-year risk of > 15% for CHD for people aged 35-74 years, without a prior history of CHD or stroke, using Framingham Heart Study functions. High risk was based on 15% 10-year CHD risk as it has been shown that the risk of combined CVD (risk of CHD plus stroke/transient ischemic attack) may be obtained by multiplying the estimated 10-year risk of CHD by 4/3 (i.e., 15% risk of CHD = 20% risk of CVD) ^16^. The probability that a risk of CHD > 15% would identify risk of combined CVD > 20% has been shown to be 91% in white people [16].

### Statistical analysis

Data were weighted to the 1999 Estimated Residential Population and 2001 Census for South Australia by region, age group, gender, and probability of selection in the household, to provide population representative estimates [17]. Data were analysed using the Statistic Package for the Social Sciences (SPSS version 15.0; SPSS, Chicago, IL). Pearson's Chi-square tests determined significant differences in proportions. Multiple logistic regression models were developed to describe associations of socio-demographic characteristics with use of i) non-specific NSAIDs (ns-NSAID), other than low-dose aspirin, ii) COX-2 inhibitors, iii) all NSAIDs, adjusted for age, sex, education level, employment status and receipt of government benefits.

## Results

The demographic profile of cohort participants has been previously published [14]. Table 1 shows the prevalence and multivariable associations for reported NSAID use in various demographic categories. NSAIDs were used by 357 (11.2%) of adults, 147 (9.8%) males and 210 (12.6%) females, and ranged from 3.6% use in 20-34 year olds to 17.9% among over 75 year olds. Among the 176 people using ns-NSAIDs, naproxen was used by 39.8%, diclofenac by 39.8%, and ibuprofen by 11.9%. Of these, there were 38 who exclusively purchased medication over-the-counter (ibuprofen 31 of 51 users; naproxen 7 of 21), i.e. 21.6% of ns-NSAID users purchased these only over-the-counter. In multivariable analyses, COX-2 inhibitors were used 3.7 times more often by those aged 75 years and older than in 35 to 54 year olds (Table 1). Rofecoxib use was reported by 31 (0.98%) people. There was no association between advancing age and use of ns-NSAIDs.

**Table 1:** Prevalence [% (n)] of non-steroidal anti-inflammatory drug (NSAID) use and multivariable adjusted odds ratios (95% confidence intervals) of NSAID use associated with demographic factors*

	ns-NSAIDs (n = 176)		COX-2 inhibitors (n = 187)		Combined (n = 357**)	
	**% (n)**	**OR (95% CI)**	**% (n)**	**OR (95% CI)**	**% (n)**	**OR (95% CI)**

**Sex**						
Male	4.8 (73)	1.0 (ref)	5.0 (76)	1.0 (ref)	9.8 (147)	1.0 (ref)
Female	6.2 (103)	1.2 (0.9-1.7)	6.7 (111)	1.5 (1.03-2.0)	12.6 (210)	1.3 (1.04-1.7)

**Age**						
20-34	3.3 (10)	0.7 (0.4-1.4)	0.3 (1)	0.13 (0.02-0.98)	3.6 (11)	0.5 (0.3-0.98)
35-54	4.8 (60)	1.0 (ref)	2.3 (29)	1.0 (ref)	6.9 (87)	1.0 (ref)
55-74	6.5 (78)	0.9 (0.6-1.4)	9.1 (109)	3.1 (1.9-5.1)	15.4 (184)	1.6 (1.2-2.3)
75+	6.7 (28)	0.8 (0.4-1.5)	11.5 (48)	3.7 (2.0-6.7)	17.9 (75)	1.7 (1.1-2.6)

**Education**						
High school or less	6.2 (100)	1.0 (ref)	7.0 (113)	1.0 (ref)	13.1 (211)	1.0 (ref)
Diploma/certificate/trade	5.4 (62)	1.0 (0.7-1.4)	5.2 (59)	1.1 (0.7-1.5)	10.4 (119)	1.0 (0.8-1.3)
University degree	3.4 (14)	0.7 (0.4-1.2)	3.6 (15)	1.2 (0.7-2.2)	6.5 (27)	0.8 (0.5-1.3)

**Employment**						
Full time	3.2 (36)	1.0 (ref)	2.2 (25)	1.0 (ref)	5.1 (58)	1.0 (ref)
Part time/casual	6.7 (35)	1.9 (1.1-3.1)	3.8 (20)	1.1 (0.6-2.1)	10.3 (54)	1.6 (1.1-2.4)
Unemployed	4.2 (3)	1.01 (0.3-3.5)	5.6 (4)	1.3 (0.4-4.1)	9.9 (7)	1.2 (0.5-3.0)
Home duties	5.2 (20)	1.2 (0.6-2.4)	6.8 (26)	0.9 (0.5-1.9)	11.9 (46)	1.2 (0.7-1.9)
Retired	7.7 (72)	2.0 (1.04-3.7)	10.5 (98)	1.2 (0.6-2.3)	18.2 (169)	1.7 (1.05-2.7)

**Government benefit ^†^**						
No	4.2 (77)	1.0 (ref)	2.8 (51)	1.0 (ref)	6.8 (125)	1.0 (ref)
Yes	7.6 (99)	1.4 (0.9-2.1)	10.3 (134)	1.9 (1.2-3.1)	17.7 (230)	1.6 (1.2-2.3)

*n = 11 missing data for income, employment, and government benefit

**6 participants were using both COX-2 and NSAIDs

**^† ^**Government benefit includes all pensions or benefits but not including family allowance.

Among those people using NSAIDs, 61% had hypertension, with 41% reported concurrent use of anti-hypertensive medication (Table 2). Thirty-one percent of people taking NSAIDs had Stage 3 or higher chronic kidney disease. Cardiac disease, hypertension and renal disease were relatively more common among people using COX-2 inhibitors than those using ns-NSAIDs, as was the use of medications to treat these conditions.

**Table 2:** Prevalence [%, (n)] of chronic conditions and medication use among people reporting NSAID use

	Reported NSAID use			Ns-NSAID			COX-2 inhibitor		
	**No**	**Yes**	**p**	**No**	**Yes**		**No**	**Yes**	

Chronic conditions, % (n)									
Joint pain									
*Diagnosed as arthritis*	23.4 (649)	70.8 (247)	< 0.01	26.9 (796)	57.5 (100)	< 0.01	25.3 (745)	83.4 (151)	< 0.01
*Not diagnosed as arthritis*	39.4 (1095)	22.6 (79)		37.8 (1117)	32.8 (57)		39.0 (1151)	12.7 (23)	
Cardiovascular disease/stroke	8.7 (240)	17.2 (60)	< 0.01	9.4 (276)	13.8 (24)	0.05	9.0 (264)	20.0 (36)	< 0.01
MI/angina	6.5 (182)	12.0 (42)	< 0.01	7.0 (208)	9.2 (16)	0.28	6.7 (198)	14.4 (26)	< 0.01
> 15% 10 year CVD risk	14.9 (324)	20.7 (56)	0.01	15.4 (355)	17.9 (25)	0.44	15.1 (348)	23.7 (32)	< 0.01
Hypertension*	40.3 (1137)	60.8 (217)	< 0.01	42.0 (1261)	52.8 (93)	< 0.01	41.0 (1226)	68.4 (128)	< 0.01
Kidney disease**	20.6 (576)	30.8 (109)	< 0.01	21.6 (644)	23.4 (41)	0.58	20.8 (616)	37.3 (69)	< 0.01

Primary care provider visits in previous year									
*0*	7.9 (219)	2.3 (8)	< 0.01	7.4 (220)	4.0 (7)	< 0.01	7.7 (226)	0.6 (1)	< 0.01
*1-4*	53.0 (1474)	29.4 (103)		51.5 (1521)	32.2 (56)		51.9 (1530)	26.0 (47)	
*≥ 5*	39.1 (1087)	68.3 (239)		41.1 (1215)	63.8 (111)		40.5 (1193)	73.5 (133)	

Medications									
Anti-hypertensives	26.6 (749)	40.9 (146)	< 0.01	27.9 (836)	33.5 (59)	0.11	27.0 (806)	47.6 (89)	< 0.01
Diuretics	3.6 (101)	9.0 (32)	< 0.01	4.1 (123)	5.7 (10)	0.31	3.7 (110)	12.3 (23)	< 0.01
ACE inhibitors	11.3 (319)	12.0 (43)	0.69	11.2 (336)	14.8 (26)	0.15	11.5 (344)	9.6 (18)	0.43
Gastro-esophageal reflux	12.3 (347)	31.9 (114)	< 0.01	14.0 (419)	23.9 (42)	< 0.01	12.9 (386)	40.1 (75)	< 0.01

*Hypertension: systolic ≥ 140 mm Hg and/or diastolic ≥ 90 mm Hg or use of anti-hypertensive medication.

**Kidney disease: GFR < 60 ml/min/1.73 m^2^, GFR > 60 and proteinuria.

Of the overall sample, n = 1697 (42%) 1819 (45%) had one or more of the identified chronic conditions where use of NSAIDs is contraindicated or were at high-risk of adverse events (i.e. cardiovascular disease, cerebrovascular disease, chronic kidney disease, hypertension, high-risk for cardiovascular disease). Among the n = 357 with NSAID use, 246 (69%) 274 (77%) had one or more contraindications, including 141 (75%) who use COX-2 inhibitors, and 109 (62%) of those taking ns-NSAIDs. If anti-reflux medications are included as a relative contra-indication, these numbers rise to 274 (77%) of all NSAID users, with 274 (84%) of COX-2 inhibitor users, and 121 (69%) of those using ns-NSAIDs, with 1819 (45%) of the population with a relative contra-indication.

NSAID use was common among people with chronic conditions (Table 3). Sixteen percent of all people with uncontrolled hypertension and 16% with kidney disease were using NSAIDs, as were 20% of people with cardiovascular disease. Around a quarter taking either diuretics (24.1%) or acid-lowering medication (24.7%) reported NSAID use. Among people with joint pain not diagnosed as arthritis NSAID use was much less common, and COX-2 inhibitor use was uncommon. People with cardiac disease, hypertension and renal disease more commonly used COX-2 inhibitors than ns-NSAIDs. People taking anti-hypertensives, diuretics and medications for gastro-esophageal reflux more commonly used COX-2 inhibitors than ns-NSAIDs.

**Table 3:** Prevalence of NSAID use [%, (n)] among people with various chronic conditions and medication use

Chronic conditions		NSAID use % (n = 357)		NSAID use (n = 176)		Cox-2 inhibitor use (n = 187)	
Joint pain	No	2.2 (23)	< 0.01	1.6 (17)	< 0.01	0.7 (7)	< 0.01
	Yes						
	*Diagnosed as arthritis*	27.6 (247)		11.2 (100)		16.9 (151)	
	*Not diagnosed as arthritis*	6.7 (79)		4.9 (57)		2.0 (23)	

Cardiovascular disease/stroke	No	10.2 (289)	< 0.01	5.3 (150)	0.05	5.1 (144)	< 0.01
	Yes	20.0 (60)		8.0 (24)		12.0 (36)	

MI/angina	No	10.6 (308)	< 0.01	5.4 (158)	0.28	5.3 (155)	< 0.01
	Yes	18.8 (42)		7.1 (16)		11.6 (26)	

> 15% 10 year CVD risk	No	10.4 (214)	0.01	5.6 (115)	0.44	5.0 (103)	< 0.01
	Yes	14.7 (56)		6.6 (25)		8.4 (32)	

Hypertension*	No	7.7 (140)	< 0.01	4.6 (83)	< 0.01	3.2 (59)	< 0.01
	Yes	16.0 (217)		6.9 (93)		9.5 (128)	

Kidney disease**	No	9.9 (245)	< 0.01	5.4 (134)	0.58	4.7 (116)	< 0.01
	Yes	15.9 (109)		6.0 (41)		10.1 (69)	
Primary care provider visits in previous year	None	3.5 (8)	< 0.01	3.1 (7)	< 0.01	0.4 (1)	< 0.01
	*1-4*	6.5 (103)		3.6 (56)		3.0 (47)	
	*≥ 5*	18.0 (239)		8.4 (111)		10.0 (133)	

**Medication use**							

Anti-hypertensives	No	9.3 (211)	< 0.01	5.1 (117)	0.11	4.3 (98)	< 0.01
	Yes	16.3 (146)		6.6 (59)		9.9 (89)	

Diuretics	No	10.7 (325)	< 0.01	5.5 (166)	0.31	5.4 (164)	< 0.01
	Yes	24.1 (32)		7.5 (10)		17.3 (23)	

ACE inhibitors	No	11.2 (314)	0.69	5.3 (150)	0.15	6.0 (169)	0.43
	Yes	11.9 (43)		7.2 (26)		5.0 (18)	

Gastro-esophageal reflux	No	9.0 (243)	< 0.01	4.9 (134)	< 0.01	4.1 (112)	< 0.01
	Yes	24.7 (114)		9.1 (42)		16.3 (75)	

*Hypertension: systolic ≥ 140 mm Hg and/or diastolic ≥ 90 mm Hg or use of anti-hypertensive medication.

**Kidney disease: GFR < 60 ml/min/1.73 m^2^, GFR > 60 and proteinuria.

## Discussion

We have shown a high prevalence of current NSAID use among groups at-risk for significant adverse drug-related events or who had major chronic conditions that are relative contraindications to NSAID use. Around three-quarters of people using NSAIDs have one or more of cardiovascular disease, hypertension or chronic kidney disease. These include over half of people reporting NSAID use with hypertension and nearly one-third of reported users had Stage 3 or higher chronic kidney disease. A fifth of all people with cardiovascular disease report NSAID use. This usage was not driven by isolation from health care services, as over two-thirds of NSAID users reported 5 or more GP visits in the past year.

Recent evidence that cardiovascular mortality is increased by some NSAIDs among people without a prior history of cardiac disease [8] highlights the importance of examining patterns of NSAID use and outcomes of use at a population level. COX-2 inhibitors can only be obtained by prescription, while some ns-NSAIDs can be purchased over-the-counter without a prescription (albeit at lower doses than possible via prescription), although this was a minority of users did this exclusively in our study. There were clear differences in the trends of use of ns-NSAIDs and COX-2 inhibitors with socio-demographic characteristics examined. COX-2 inhibitor use was seen more commonly in people with cardiac disease, hypertension and renal disease compared with ns-NSAID use. People on medications to treat hypertension or cardiac disease more commonly used COX-2 inhibitors than ns-NSAIDs. This suggests different considerations are operational for both patients and their doctors across clinical and demographic categories in determining NSAID use. Roughead et al found that following the introduction of COX-2 inhibitors onto the Australian market there was a large increase in overall NSAID use, with subsequent decline when rofecoxib was withdrawn. Prescribing trends were similar in people also taking diabetes medications or ACE inhibitors to the rest of the population [13]. While our data are not conclusive, it is consistent with the notion that putative GIT safety is taken into consideration when prescribing whilst cardiac disease, renal function and blood pressure have less influence on management decisions [13,18]. As COX-2 inhibitors are a prescription only medication, and most ns-NSAID use was via prescription, our results suggest that the risks of COX-2 inhibitors for cardiac disease, hypertension or renal disease are regarded as acceptable or are ignored by many clinicians. Rofecoxib was withdrawn early in the study period and few people reported its use. Thus our conclusions are driven by NSAID use other than that of rofecoxib.

Although NSAIDs are some of the most commonly used medications, their safe and effective management is complex. Shorter clinic visits have been associated with unnecessary NSAID prescribing which was related to a lesser likelihood that relative contraindications to NSAID therapy were assessed during these brief visits [19]. This problem may be confounded when caring for socially disadvantaged patients with complex biomedical and psychosocial problems and multiple barriers to care placing great demands onto a 10-15 minute primary care visit [20]. We found significantly increased use of NSAIDs among people receiving government benefits and part-time workers in the multivariable analyses, which may reflect social disadvantage.

Studies suggest increased clinician knowledge has minimal [20] or no effect [21] on NSAID prescribing. In one study, no association was found between knowledge of NSAID therapy and suboptimal management [20]. A targeted education intervention had only a small effect on prescriber's actions in NSAID use in one study [21]. Other explanations may include differences between doctors in their perceptions of the importance of treatment risks or in the expected benefits of drug therapy [22], particularly given the conflicting publicity surrounding such issues as cardiovascular risk associated with NSAID use. For a variety of reasons clinicians may not be aware of contraindications to NSAID use in individual patients, for example patients may see a number of general practitioners and specialists for their care. Patient attitudes and knowledge as well as willingness to ask questions of clinicians will have impact on NSAID use. It may be that some NSAID users are well aware of the potential harms but use NSAIDs because they get relief from pain that they can't obtain through other means. However, many patient's lack of awareness of NSAID toxicity with US data demonstrating that among patients with knee osteoarthritis (OA) 54% were unaware of NSAID toxicity and 80% unaware of COX-2 toxicity [23]. These results are particularly problematic given the wide availability of over-the-counter (OTC) NSAIDs. In contrast, a study of patient preferences in older patients with knee OA determined that most were willing to forgo treatment effectiveness for a lower risk of adverse effects [24]. A recent review of published studies in OA concluded that for every ten patients treated with paracetamol instead of a NSAID for OA, only one patient would discontinue treatment due to lack of efficacy, implying that 9 out of ten patients would continue with paracetamol [25]. Given the scope of the problem identified in our study, efforts to improve the health literacy of the community [26] have the potential to have a substantial impact on the burden-of-illness and costs of NSAIDs. The more widespread use of tools or decision-aids to assist patient decision-making may be a potential solution [27]. However research to assess the impact of sophisticated decision-support software which could present individually calculated risks against likely pain benefits from NSAIDs to patients and doctors is needed.

Our study is limited to one urban region and it is possible substantial regional differences in patterns of medication use may occur across Australia. Recent US data has shown considerable geographical variation in prescription of NSAIDs to elderly people with chronic renal disease [28]. However, other Australian studies have found similar patterns in national and regional data, including one recent study which found that use of ns-NSAIDs, COX-2 inhibitors and paracetamol overall among concession beneficiaries was comparable in Australia and Queensland [29]. Our data on medication use relies on participants bringing their medication to the study clinic. However, both prescription-based data and general practitioner databases may underestimate the use of NSAIDs as unreported OTC usage is common. Similarly general practitioner databases may also underestimate NSAID use, as patients may not report use of OTC medications to doctors. In one US study, almost one in five respondents did not report use of an NSAID to clinical staff, including 8% who reported daily use. For 22% of respondents, they did not think the medications were important enough to list, while 30% cited the fact that the drugs were not prescribed by a physician [30]. This reflects a common misperception that these medications are insignificant or benign when actually their chronic use, particularly among the elderly and those with conditions such as arthritis, is linked to serious and potentially fatal adverse effects [31].

Our survey was limited to households with listed telephone numbers, but as 97% of the households in the region had telephones during the study period, and the demographic characteristics were representative of the population of profile of Adelaide overall [14], the extent of any bias is likely to be small. Underestimation of cardiac history from self-report in our sample may also have attenuated the relationship with NSAIDs. However, studies from community populations have found self-reported cardiac events and stroke to be accurate [31]. Framingham risk equations tend to overestimate risk in the affluent elderly and underestimate in the poorer young. Loss to follow-up may also bias our findings. The strength of this study is the large representative population sample with known probability of selection, measurements of other known chronic disease risk factors, and low drop-out rate in follow-up, especially in people over 45 years who are more likely to be at risk for chronic disease and use of NSAIDs, and that participants brought in all medications they were taking (including both prescribed and over-the-counter) and that usage was clarified at the clinic visits.

## Conclusions

There is a high prevalence of current NSAID use among groups at-risk for significant drug-related adverse events or who have major chronic conditions that are relative contraindications to NSAID use. Assessment of absolute risks regarding cardiovascular and kidney disease need to take into account use of medications such as NSAIDs. The potential to make a substantial impact on chronic disease burden via improved use of NSAIDs is considerable.

## Competing interests

RJA has received speaker's fees from Glaxo-Smith Kline.

## Authors' contributions

RJA took primary responsibility for authoring the manuscript, SLA and TKG undertook most of the data analysis, CLH conceived the analysis and contributed to writing the manuscript, AWT and DHW had the major role in design and conduct of the study and contributed to writing of the manuscript.

All authors have read and approved the final manuscript.

## Pre-publication history

The pre-publication history for this paper can be accessed here:

http://www.biomedcentral.com/1471-2296/12/70/prepub
